# Arming the troops: Post-translational modification of extracellular bacterial proteins

**DOI:** 10.1177/0036850420964317

**Published:** 2020-11-04

**Authors:** Suzanne Forrest, Martin Welch

**Affiliations:** Department of Biochemistry, University of Cambridge, Cambridge, UK

**Keywords:** Post-translational modification, protein secretion, bacterial pathogens, bacterial virulence factors, proteomics, methylation, acetylation, glycosylation, lipidation

## Abstract

Protein secretion is almost universally employed by bacteria. Some proteins are retained on the cell surface, whereas others are released into the extracellular milieu, often playing a key role in virulence. In this review, we discuss the diverse types and potential functions of post-translational modifications (PTMs) occurring to extracellular bacterial proteins.

## Introduction

**Figure 1.**
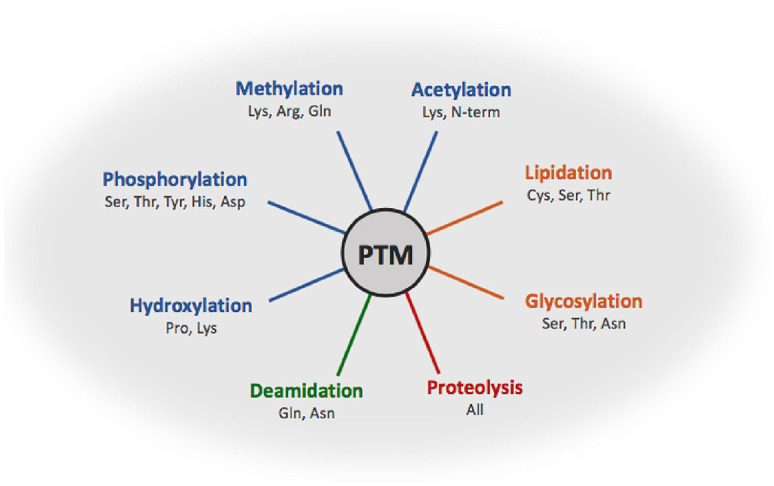
An overview of the most common post-translational modifications in bacteria, showing the amino acid side chains which are most frequently modified. PTMs are grouped by colour according to their type; small chemical (blue), complex molecule (orange), protein cleavage (red) and amino acid side chain modifications (green).

Until relatively recently, the nature and effects of post-translational modifications (PTMs) were principally thought to be restricted to eukaryotic systems. However, PTM in prokaryotes is now appreciated to be just as important and diverse as it is in eukaryotes.^1^ The ever-expanding catalogue of bacterial PTMs ranges from methylation and phosphorylation of residues to the addition of complex moieties including lipids and glycans (Figure 1).^1^ These modifications can have profound effects on proteins, altering their conformation, activity, stability and localisation, as well as interactions with other molecules.^2^ The specific purpose of PTMs is not always clear, although they have been shown to modulate and mediate key biological processes, including central metabolism, signal transduction and virulence.^3,4^ Not surprisingly, many reversible PTMs also appear to be involved in mediating rapid responses to environmental stimuli.^5^

Historically, the earliest studies focused on the PTMs associated with individual proteins, and often just on the impact of modifications at specific sites. However, advances in proteomic technologies have driven a surge in the number of large-scale global modification studies for a wide range of bacterial species.^5,6^ Despite this, there are a number of clear limitations to the technology. High levels of purity and quantity of protein are generally necessary for sufficient sequence coverage and resolution to identify PTMs using mass spectrometry-based approaches.^7^ This can be bolstered through selective enrichment of post-translationally modified proteins (e.g. using antibody-based approaches) prior to mass spectrometric analysis, although this is predicated on a high degree of specificity and high binding capacity of the antibodies employed.^8^ Perhaps a more pervasive issue is that the greater the number of different types of PTM to be identified, the larger the bioinformatic search space required. This is due to an iterative search mechanism which attempts to identify the presence or absence of each PTM on every modifiable residue. This ultimately increases the rate of false discoveries.^9^ There are also limits on the mass shift window authorised for searches, so larger modifications such as glycosylation are frequently excluded.^5^ Consequently, independent approaches are often required to validate these “high throughput” technologies.^2,10^ These include structural and functional studies, although as always, these too can be challenging.^11^

Although it is now widely accepted that PTMs occur in bacteria, most studies have focused on cell-associated protein modifications, and relatively few have considered the modifications associated with proteins secreted into the extracellular milieu. Indeed, most culture supernatants are usually removed prior to mass spectrometric analysis.^6,12–14^ However, recent work has revealed a wealth of unexpected PTMs associated with extracellular bacterial proteins, including phosphorylation, methylation, acetylation, proteolytic processing, glycosylation and lipidation.^15^ In this review, we assess the diversity and likely function(s) of PTMs associated with extracellular bacterial proteins.

## Phosphorylation

Protein phosphorylation is a ubiquitous and abundant PTM, usually associated with intracellular signal transduction.^16,17^ The attachment and removal of phosphoryl groups on amino acid side chains is catalysed by kinases and phosphatases, respectively.^2^ Serine, threonine and tyrosine are commonly phosphorylated in eukaryotes, whereas histidine and aspartate phosphorylation are thought to be more prevalent in prokaryotes, although this is disputed.^18,19^ Microbial phosphoanhydrides (Asp-P) and phosphoramidates (His-P) are more labile than the phosphoesters that form with Ser, Thr and Tyr side chains, and this makes them more difficult to detect, especially if samples are exposed to acidic conditions during preparation.

*Mycobacterium tuberculosis* (Mtb) is a global health burden. The organism is now strongly resurgent, partly due to widespread multi-drug resistance. Effector proteins are secreted directly from the donor Mtb cell cytoplasm to the recipient (host) cell cytoplasm through the multiprotein ESX-1 Type VII secretion system (T7SS). Not only are a sizeable number of these structural proteins phosphorylated; so too are some of the virulence factors which pass through this translocon, such as the immune-triggering proteins EsxB and PtpA.^18^ PtpA is a tyrosine phosphatase that is secreted into macrophages following phagocytosis of the bacterium, causing inhibition of phagosome maturation and thus promoting bacterial survival. PtpA is phosphorylated at several S/T/Y residues, and this has been shown to control its activity and secretion.^20^ Interestingly, phosphorylation of multiple virulence factors is up-regulated in the hypervirulent Mtb Beijing isolate.^18^ Phosphorylation is also used as a regulatory mechanism to temporally control different stages of infection by other bacterial species. The *Helicobacter pylori* cytotoxin CagA is phosphorylated on tyrosine by host membrane-associated Abl1 and/or Src family kinases. Interestingly, CagA phosphorylation is stimulated by another *H. pylori* secreted product, vacuolating toxin (VacA). This effector is itself phosphorylated but also promotes the Src-mediated phosphorylation of CagA.^21,22^ CagA and VacA effectors are involved in the early stages of gastric colonisation and are modified following injection into the epithelial cells lining the stomach.^23^

Not all secreted effector proteins are phosphorylated by bacterial kinases. For example, host kinases can phosphorylate some of the effector proteins produced by enteropathogenic *Escherichia coli* and *Citrobacter rhodentium*.^24^ At least 4 proteins secreted by the T2SS and T3SS of Chlamydial species are similarly modified, including TarP and TeP, which facilitate entry into the mucosal epithelia.^25^ Bacteria can also hijack and control host systems through PTM. The Dot/Icm (T4SS) of *Legionella pneumophila* translocates over 300 effectors, including a kinase, LegK, which can phosphorylate host proteins to interfere with normal cell functioning.^26^

Elastase (LasB) secreted by *Pseudomonas aeruginosa* has also been shown to be phosphorylated; indeed, 19 phospho-residues have been identified in the secreted form, whereas only non-phosphorylated LasB appears intracellularly.^27^ The biological role(s) of this phosphorylation have not been elucidated, although it is possible that the modification targets the enzyme for secretion. Indeed, some 28 phospho-exoproteins with a wide range of functions and degree of modification were identified in one study of strain PA14. This suggests that there may be numerous roles for phosphorylation, particularly in *P. aeruginosa* virulence.^28^

Surface-exposed bacterial proteins are also modified in many organisms. For example, flagella proteins from several *P. aeruginosa* strains are S/T/Y phosphorylated and this modification often occurs at a very specific growth stage.^29^ Surprisingly, one of the main growth phase-dependent flagella PTMs (phosphorylation of the FliC N-terminus) does not affect swimming motility, but instead increases Type II Secretion System (T2SS) activity whilst concomitantly decreasing biofilm formation.^30^ Similarly, phosphorylation of the *Neisseria gonorrhoeae* type IV pilus protein, PilE, at Ser68 also has little apparent effect on the motility-related function of the protein, although it does influence the morphology of the pilus, and consequently, antigenic variation.^31^ Outer membrane proteins (OMPs) from *Klebsiella pneumoniae, H. pylori* and *Shigella flexneri* are also multi-phosphorylated, although the function of these PTMs remains unclear.^32,33^

## Methylation

Methylation is well characterised in eukaryotes, notably the methylation of histones to control gene transcription,^34^ but not well studied in bacteria. It involves the addition of up to two or three methyl groups to the side chain or terminal amine of arginine or lysine, respectively. Glutamine and glutamic acid residues are also modified, but to a lesser extent.^1^ S-adenosyl methionine (SAM) acts as a methyl donor, working in concert with methyltransferase enzymes to catalyse these reactions.

The *P. aeruginosa* secreted virulence factors CbpD (chitin binding protein) and elastase are methylated at several lysine residues. However, these lysines are methylated to different degrees, with mono-, di- and tri-methylated forms of the same lysine present. This indicates that methylation can be highly variable, even for the same protein.^27^ Variations in side chain methylation are also seen in the Gram-positive organism *Clostridium thermocellum.* This bacterium degrades cellulose by secreting a complex of different proteins, known as the cellulosome. CipA, a cellulosome structural protein, is methylated at Glu1267 and trimethylated at Lys80 and Lys663, although the protein is also secreted in an unmodified form. Contrastingly, the cellulolytic CelK enzyme is consistently di-methylated at Lys652 under different experimental conditions, suggesting that certain residue modifications are invariable. This PTM is postulated to promote protein flexibility, whereas methylation of glutamic acid may aid cellulosome localisation.^10^

Studies closely scrutinising secreted protein methylation are limited. However, outer membrane proteins have been studied in greater detail. OMP 32 of *Leptospira interrogans* undergoes extensive but irregular methylation. Eleven glutamic acid residues are variably modified in response to mammalian host signals.^35,36^ This leads to OMP phase variation and reduced recognition by the host immune system. Lysine methylation also alters the antigenicity of *Rickettsia* OMPs.^37^ Two different lysine methyltransferases modify OmpB from *Rickettsia prowazekii*, with one enzyme specifically catalysing tri-methylation at consensus sequences.^38,39^ Interestingly, the overall number of methylated lysine residues correlates with virulence in this strain.

One of the earliest observations of methylation as a PTM occurred in 1959 during an investigation of flagella structure and function.^40^ Over half a century later, investigations have now revealed methylation of flagella proteins in a range of species.^41,42^ Flagellin methylation by FliB, a component of the *Salmonella enterica* serovar Typhimurium flagella machinery, is necessary for swarming motility and virulence^43–45^ and orthologous methyltransferases produced by other members of the Enterobacteriaceae are encoded in flagellin methylase island loci (FMIs).^42^ In addition to flagella, surface-associated pili can also be methylated. The pre-pilin peptidase (PilD) of *P. aeruginosa* acts as a dual modifier, by cleaving the signal peptide and then methylating the N-terminal phenylalanine of mature Type 4 pilus subunits. Methylation happens prior to pilus assembly and is dependent on the binding of zinc as a cofactor.^46^ This processing also occurs in *Neisseria meningitidis*, although methylation is not a prerequisite for proper pilus assembly and the true function of this PTM remains unknown.^47,48^

EF-Tu is an elongation factor that delivers charged tRNA to ribosomes in the cytoplasm. It is also transported to the bacterial surface where it “moonlights” as an environmental sensor and aids adherence to epithelial cells in many species.^49^ A protein is described to “moonlight” when it has additional function(s) that are not relevant to its primary role within the cell. *P. aeruginosa* EftM exclusively tri-methylates EF-Tu on Lys5. This modification does not impact the canonical function of the protein in translation, but does mediate bacterial attachment to the respiratory epithelia by mimicking phosphorylcholine (a component of the platelet activating factor).^50,51^ Methylation is a prerequisite for infection, and deletion of *eftM* decreases *P. aeruginosa* pathogenicity. Interestingly, EftM is thermoregulated, displaying increased stability at 25°C. This may suggest that methylation is critical in the early stages of infection or for survival in non-host environments.^50^ This enzyme is well-conserved throughout the *Pseudomonas* and *Vibrio* genera and methylation of EF-Tu has proven indispensable for many pathogens.^51,52^

## Acetylation and succinylation

Acetylation predominantly occurs on the ε-amine of lysine side chains (Nε-acetylation) and at N-terminal amino acids (Nα-acetylation) in a co- or post-translational fashion.^1,53^ This type of acylation can also occur on the side chains of serine, threonine and tyrosine (O-acetylation).^2^ Acetylation can occur enzymatically via acetyltransferases (with acetyl groups also removed via the action of deacetylases) or non-enzymatically. Both pathways require an acetyl donor, commonly acetyl-CoA or acetyl-phosphate.^1,54^

Despite being proven to impact upon virulence in several species, the biological significance of acetylation of extracellular proteins is not well defined.^28,55–57^ Acetylation of extracellular bacterial proteins increases as cultures approach and enter the stationary phase of growth. This indicates that acetylation may impact upon protein stability, perhaps circumventing the unnecessary use of scarce resources to replenish vital proteins.^5^ The protein acetyltransferase (Pat) and deacetylase (CobB) of *S. enterica* serovar Typhimurium are involved in cell survival during growth following acidic stress, invasion of the host and replication within macrophages. *S. enterica* serovar Typhimurium mutants that are unable to acetylate proteins show reduced host inflammation, although how this relates to specific virulence factors is currently unclear.^58^

In a study of the *P. aeruginosa* strain PA14 intracellular lysine acetylome, 522 modified proteins were identified. Additionally, acetylation was enhanced when citrate was used as the sole carbon source.^59^ Notably, in addition to identifying many acetylated intracellular proteins, several *P. aeruginosa* virulence factors (some previously identified as methylated, such as CbpD and LasB) were also acetylated, including protease IV, haemolysin, exotoxin A and several components of the T6SS.^27,57,59^

Proteins involved in central metabolism are the main targets of acetylation in Mtb. For example, malate synthase G (GlcB) is a component of the glyoxylate shunt. However, GlcB is also secreted in an acetylated form to the surface of the cell, enhancing bacterial adherence to the lung epithelium. Indeed, a further 45 secreted acetylated proteins from Mtb have been identified. Multi-acetylation of heat shock protein X (HspX) inhibits the host immune response and has been linked to the latency of Mtb infections.^60^ ESAT-6 (6 kDa Early Secreted Antigenic Target) is inconsistently acetylated at the N-terminus, preventing protein-protein interactions with its cognate chaperone CFP-10.^61^ ESAT-6 is able function alone or in complex with CFP-10 to modulate the host immune response, and therefore the purpose of acetylation-driven segregation of these proteins is unclear.^62^ Other members of the ESAT-6 family also undergo Nα-acetylation, including EsxN, EsxO, EsxI and EsxA.^63–65^ Dephosphorylation of host proteins by PtbB is also critical for Mtb infection. The phosphatase activity of PtbB is controlled by acetylation/succinylation of Lys224, which is found in the lid region that covers the active site. This PTM therefore serves as a negative regulator of enzyme activity.^65^

Succinylation is another form of acylation alongside acetylation, malonylation, propionylation, butyrylation and crotonylation. Although the identification of succinylated proteins is a relatively recent area of research, the overlap between acetylation and succinylation in the secretome of PA14 is significant,^66^ with around 41% of succinylation sites also susceptible to acetylation.^59^ The functional significance of succinylation remains unclear, although the presence of succinyllysine within the pro-peptide of LasB indicates a potential role of this PTM in protein maturation or stability. Moreover, the number of acetylated/succinylated lysine residues in LasB increases upon secretion.^27^ Global profiling of succinylated proteins in PA14 identified seven sequence motifs that may direct modification, with the same signatures also evident in *Vibrio parahaemolyticus* and Mtb.^59,66,67^ This suggests that succinylation of secreted proteins may be a more widespread PTM than previously thought.

## Proteolysis

Proteolytic cleavage of proteins is a common and irreversible PTM. Endoproteases cleave the polypeptide chain at specific residues within sequence motifs, whereas exoproteases cleave the N- and C- termini.

Many extracytoplasmic proteins are transported across the cytoplasmic membrane (CM) through the sec translocation machinery, guided by an N-terminal signal peptide.^68^ In Gram-negative bacteria, additional secretion systems are employed to further transport proteins from the periplasm to outside of the cell.^69^ Inhibition of the type 1 signal peptidases that cleave signal peptides results in the accumulation of unprocessed proteins in the cytoplasm and ultimately, cell death.^70,71^ Hidden Markov models can accurately predict signal peptide sequences, although different algorithms are necessary for Gram-positive and Gram-negative bacteria.^72^ AXA motifs are common at the N-terminal cleavage site,^2,73^ but this can vary greatly between species.^74^ Somewhat controversially, in a study of Mtb secreted proteins, only 16% of secreted proteins had a cleaved signal peptide; this has also been seen in *Bacillus subtilis*.^72,75^ To our knowledge, the reason(s) for these observed discrepancies in the cleavage of signal peptides have not been investigated.

The substrates of the Por (type 9 secretion system, T9SS) of periodontitis-associated *Porphyromonas gingivalis* contain conserved C-terminal domains (CTD) that are essential for secretion. The principal virulence factors translocated through the T9SS are cysteine protease gingipains.^76^ Cleavage of the gingipain CTDs by PorU and PorZ following protein folding allows secretion through the outer membrane (OM) and subsequent glycosylation.^77–80^ Current data implicate the tertiary structure of the CTD as being the key signal for secretion,^80,81^ although some conserved sequence motifs have also been identified.^82^*Bordetella* filamentous haemagglutinin (FHA) also harbours a cleavable CTD. FHA is retained on the cell surface *via* anchoring of the CTD within the FhaC transporter.^83^ Cleavage of the CTD releases FHA from the surface, allowing passage of full-length unprocessed FHA through FhaC to act as a transmembrane sensor.^84^ This intriguing interplay between the two forms of FHA is tightly-regulated, ensuring successful colonisation and maintenance of infection.

**Figure 2.**
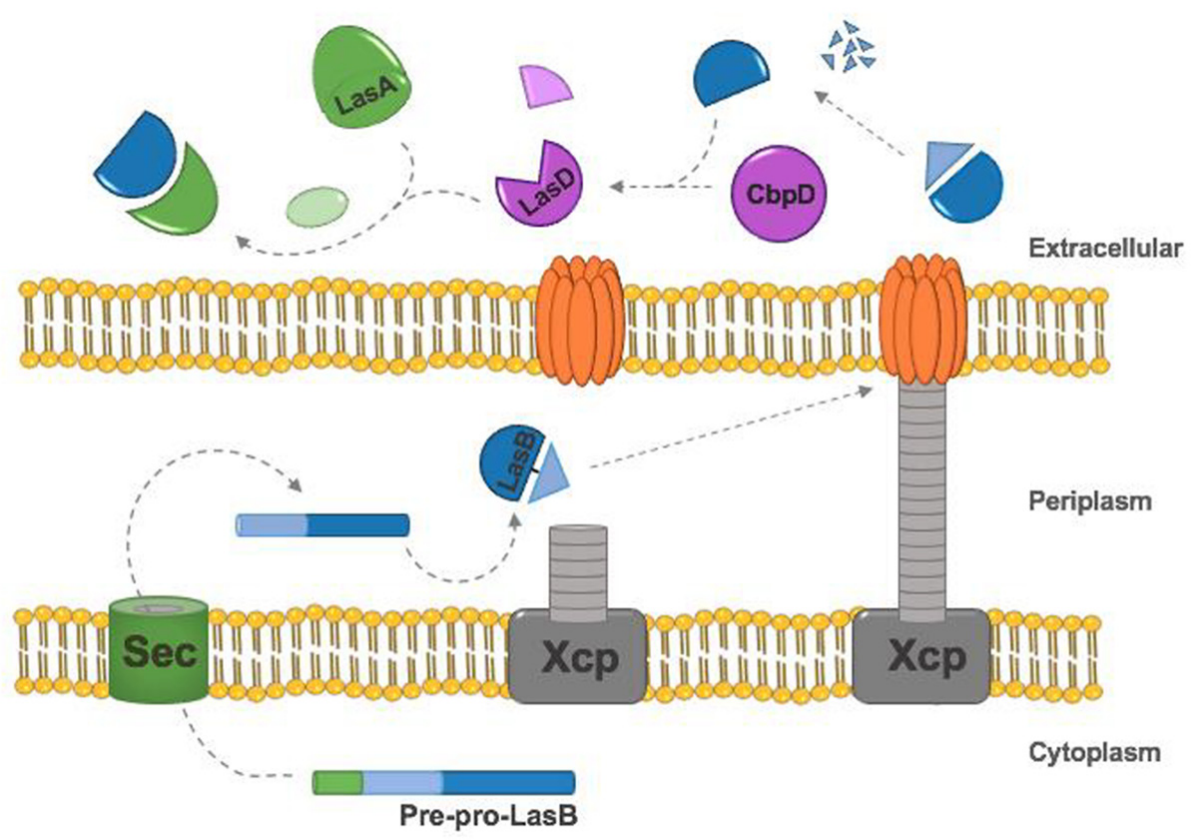
*Pseudomonas aeruginosa* LasB secretion pathway and subsequent proteolytic cleavage cascade. The pre-pro-protein is targeted to the periplasm through the sec translocon via the encoded signal peptide. The protein is then folded, and the pro-domain is cleaved, remaining non-covalently bound to the mature protein. Following secretion via the T2SS Xcp machinery, the pro-domain is degraded and a cleavage cascade begins. Mature LasB cleaves CbpD into LasD, which then activates LasA by proteolysis. Mature LasA enhances the elastolytic activity of LasB.

An elegant cleavage cascade which activates several virulence factors has been highlighted in *P. aeruginosa* (Figure 2). Elastase is synthesised as a pre-pro-protein (53 kDa) which is cleaved and transported through the CM and OM by targeting and sequential cleavage of the pre- and pro-domains, respectively. The pro-domain undergoes autoproteolytic cleavage post-folding in the periplasm but remains non-covalently linked to the mature protein (33 kDa) where it inhibits intracellular protease activity.^85^ Both domains are then secreted through the Xcp T2SS machinery and the pro-domain is subsequently degraded. A similar secretion pathway can be seen in the production of subtilisin by *Bacillus subtilis*.^86^ Extracellular LasB processes CbpD at the N-terminus, yielding LasD, which in turn, cleaves LasA into its mature staphylolytic form.^85,87,88^ The processing of these enzymes works as a positive feed-forward mechanism because LasA further enhances the elastolytic activity of LasB.^27,85^ LasB also activates leucine aminopeptidase by cleaving the C-terminal pro-sequence, which contains an active site-inhibitory lysine residue. Substitution of this C-terminal lysine with an acidic alternative results in leucine aminopeptidase activation without the need for LasB processing.^89^

Some cleavage events produce multiple functional products. For example, autolysin synthesised by *Staphylococcus aureus* is cleaved at four locations to produce functionally distinct extracellular hydrolases, an amidase and a glucosaminidase.^68^ In several diverse pathogens, EF-Tu is also cleaved into fragments which are then expressed on the cell surface. Here they act as adherence factors, binding a range of host substrates and mediating colonisation of different niches.^52^

## Glycosylation

Glycosylation involves the covalent attachment of a carbohydrate to the amide group of asparagine (N-linked) or the hydroxyl group of serine or threonine (O-linked).^90–95^ Attachment of glycans is a multi-step process involving different enzymes,^96^ which are commonly encoded within gene clusters with their substrates.^42^ Promiscuous glucosyltransferases generate extensive variability in the glycosyl moieties of modified proteins.^97,98^ Two main glycosylation pathways are used in bacteria, either *via* the *en bloc* transfer of glycan chains (preassembled on lipid carriers) to proteins, or sequential attachment from nucleotide-activated sugars.^99^

There are few examples of bacterial glycoproteins which are fully secreted into the extracellular milieu; most such modified proteins remain attached to the cell.^100–102^ The best-studied glycoproteins are flagellins.^41,103–105^*Campylobacter jejuni* flagella are N-glycosylated by PglB with a nine-carbon pseudaminic acid, which transfers glycan moieties *en bloc* to proteins at the sequon D/E-X-N-X-S/T (X≠Pro),^106,107^ although some exceptions to this rule have been found.^108^ Functionally, glycosylation may have multiple roles. For example, adherence of *Clostridium difficile* in the human gut is dependent on N-acetyl-glucosamine glycosylation of flagellar proteins. This glycan-induced switch to a more sessile mode aids biofilm formation.^109^ Flagellar glycosylation may also play a protective role. For example, the flagella of *Burkholderia cepacia* are modified at >10 sites, and these modifications are required for auto-agglutination, resistance to acid, and blocking of toll-like receptor 5 recognition.^110–112^

Many Pseudomonads encode a genomic glycosylation island as part of the flagella regulon.^98,103^*P. aeruginosa* produces two types of flagellin proteins which are distinguished by their antigenicity. Both types are glycosylated, although they are modified by different machinery encoded by different gene clusters. O-linked glycosylation occurs at Thr189 and Ser260 found in the central surface-exposed domain of each A type flagellin unit.^98^ Interestingly, type B flagellins are also glycosylated twice, at Ser191 and Ser195.^103^ Whilst the specific role of O-linked flagella glycosylation is unclear in *P. aeruginosa*, glycosylation of flagella in *Pseudomonas syringae* is involved in bacterial recognition by plants, and can shape host specificity.^113^

O-glycosylation is commonly used to protect surface-associated proteins from degradation by extracellular proteases. *H. pylori* alpha and beta ureases, *Microcystis aeruginosa* microcystin-related protein C, and *Streptococcus mutans* binding protein Cnm all undergo O-glycosylation to increase protein stability.^97,114,115^ This is also the case for EmaA, the only *Aggregatibacter actinomycetemcomitans* autotransporter adhesin which is currently known to be glycosylated. The other adhesins, ApiA and AaE, are unmodified.^116^ Additional autotransporter proteins and adhesins from unrelated bacteria are also secreted as glycoproteins, including AtaC from *Actinobacillus pleuropneumoniae*, and the self-assembling TibA from *E. coli*.^117,118^ Glycosylation of TibA by TibC enhances stability and adhesion to epithelial cells, although the modification is not known to affect invasion or aggregation of *E. coli*, unlike its other autotransporter glycoproteins.^117^

Adherence-promoting surface fimbriae can also be modified by O-linked glycosylation. For example, unmodified fimbria-associated glycoprotein (Fap1) from *Streptococcus parasanguinis* mediates cell adhesion, whereas following glycosylation, the protein becomes essential for biofilm development.^119^ Variable glycosylation of Gram-negative pili is also widespread.^48,99^ Two of the five type 4 pili produced by *P. aeruginosa* are modified by PilO *via* the addition of O-antigen or polymers of D-arabinofuranose. The even distribution of glycans on the pili fibrils confers protection against infection by bacteriophage which target pili as receptors for adsorption.^120^ In true tit-for-tat competitive style however, phage can mutate their tail proteins and adapt their specificity to re-sensitise against the bacterial host.^121^ Overall glycosylation of surface proteins contributes towards enhanced protection and stability; improved adherence, invasion and immune stimulation; and increased uptake of DNA.^107,122^

## Lipidation

**Figure 3.**
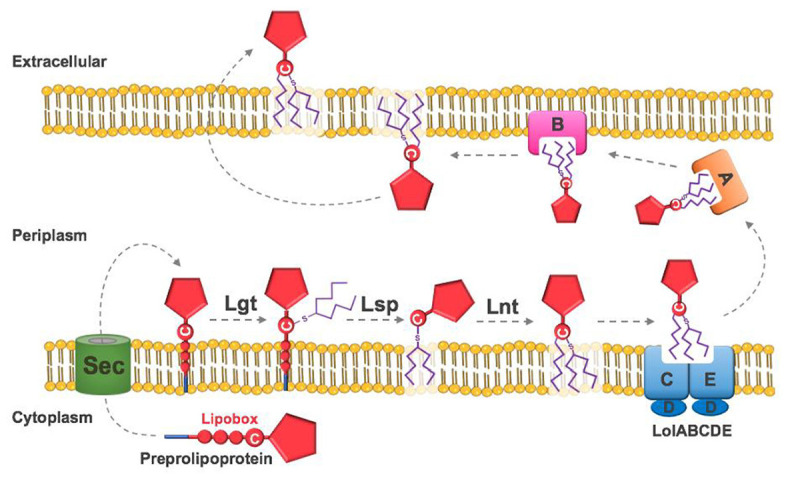
Biosynthesis pathway of lipoproteins in *E. coli*. Preprolipoproteins are synthesised in the cytoplasm and targeted through the sec translocon to the periplasm via their N-terminal signal peptide. Diacylglycerol transferase (Lgt) transfers diacylglycerol to the thiol group of the last cysteine in the four-residue lipobox motif. After this, lipoprotein signal peptidase (Lsp) cleaves the signal peptide immediately before the derivatised cysteine and apolipoprotein N-acyltransferase (Lnt) transfers another acyl group to the cysteine N*α* moiety. The mature lipoprotein is recognised by the Lol ABC transporter, which transfers the lipoprotein to the outer membrane.^132^

Lipidation is another complex PTM, typically involving the addition of two or three lipids to the N-terminal cysteine of lipobox sequence motifs.^123,124^ The lipid moieties act as a surface anchor and tend to reflect the fatty acid composition of membrane phospholipids. This contributes to the significant antigenic variability of lipoproteins between- and within-species.^125,126^ The lipoprotein biosynthesis pathway involves up to three sequential enzymatic reactions (Figure 3).^127,128^ Interestingly, and despite not encoding a homologue of the final N-acyltransferase enzyme (Lnt), many low G+C Gram-positive organisms are still able to produce triacylated lipoproteins.^129–131^

Lipoproteins play a substantial role in bacterial growth and pathogenicity. The role(s) played by lipidation are nothing if not diverse. For example, lipidation enhances Streptococcal adherence to endothelial cells, and loss of a single *S. sanguinis* lipoprotein (the metal ion transporter, SsaB) drastically decreases the ability of the organism to cause endocarditis.^133–135^ Lipidation is also known to affect the flagellar-driven motility of *C. jejuni* in the gut^136^ and significantly impacts the virulence of *Enterococcus faecalis*. Somewhat unexpectedly, loss of the lipidation enzymes also enhances *E. faecalis* growth under stressed conditions.^137^ In contrast, the growth of *S. aureus* during nutrient limitation has been reported to be dependent on lipidation.^138^ Interestingly, *S. aureus* triacyl-lipoprotein production is strongly-dependent on environmental parameters and growth phase, with diacyl forms dominating in high-stress conditions.^139^ Indeed, the role of the third fatty acid has been questioned in Gram-positive organisms as triacylation is generally thought to target lipoproteins across the outer membrane.

*N. meningitidis* surface antigen Factor H binding protein (FHbp) is normally tri-palmitoylated *in vivo*. In contrast to previous reports, deletion of Lnt (which adds the third palmitoyl group) does not prevent the surface expression of diacylated FHbp.^140,141^ However, the outer membrane Lol transporter has a higher affinity for the triacylated form and accumulation of diacylated FHbp results in negative feedback and reduced overall FHbp synthesis. It is possible that production of both the di- and tri-acylated FHbp isoforms may confer a fitness advantage in terms of antigen recognition, or that sole production of the diacylated FHbp isoform leads to a fitness disadvantage (e.g. *via* elevated antibiotic sensitivity due to increased membrane permeability^142^).

Surface dissociation of di- or triacylated lipoproteins during infection activates host TLR2/6 or TLR2/1 heterodimers respectively, which drives inflammation.^132,143,144^ In *S. aureus*, lipoprotein release is mediated by quorum sensing-controlled expression of surfactant-like phenol-soluble modulins (PSMs). These small peptides not only promote the release of lipoprotein-containing membrane vesicles during cell turgor in hypotonic conditions, but themselves act as potent virulence factors.^145^ Another virulence factor that is regulated by lipidation is the secreted pore-forming toxin, haemolysin, from *E. coli.* This toxin induces apoptosis in target host cells and is activated prior to export by the addition of fatty acids to two internal lysine residues.^146,147^ Many other species also use lipidation to regulate haemolysin activity e.g., palmitoylation of *B. pertussis* haemolysin.^148,149^

The localisation of virulence factors within host cells is important for their biological function. The HopZ family of T3SS effectors produced by *P. syringae* are targeted to the plant host plasma membranes *via* myristoylation.^150,151^ Proper targeting of these effectors causes programmed cell death through immune modulation.^152^ In a remarkable example of molecular subterfuge, some pathogens encode a CAAX motif on their secreted proteins; this motif is lipidated by host acyltransferases, thereby targeting the effectors to distinct organelles. Palmitoylation by host cell enzymes is also essential for the proper localisation, stability and activity of the polyclonal B-cell mitogen, PrpA, produced by Brucella species.^153^ Indeed, this may be a common feature of many intracellular pathogens, since secreted proteins produced by *L. pneumophila* and *S. enterica* serovar Typhimurium have also been shown to hijack the host cell machinery during targeting, thereby presumably conserving costly resources.^154–156^

## Concluding remarks

Since the start of the 21st century, considerable gains have been made in the field of prokaryotic PTM research. However, until recently, the importance of extracellular protein PTMs has been largely underestimated. Nonetheless, many secreted proteins from diverse bacterial species have been shown to be modified in a variety of ways. Although some of these modifications are proving essential for bacterial physiology and virulence, the purpose of many others remains unresolved. What is clear though, is that PTM is not a singular mechanism of control, and global studies of bacterial proteomes have shown considerable overlap between modifications.^2,157^ This notwithstanding, the era of defining the type and extent of such PTMs now seems to be drawing to a close; the challenge for the future generation will be in defining the biology underpinning these modifications.
